# Cinnamaldehyde as antimicrobial in cellulose‐based dental appliances

**DOI:** 10.1111/jam.15283

**Published:** 2021-09-22

**Authors:** Sarah Worreth, Vivien Bieger, Nadja Rohr, Monika Astasov‐Frauenhoffer, Tino Töpper, Bekim Osmani, Olivier Braissant

**Affiliations:** ^1^ Department of Biomedical Engineering University of Basel Allschwil Switzerland; ^2^ IUT Nancy‐Brabois Université de Lorraine Lieu‐dit Le Montet Villers‐lès‐Nancy France; ^3^ Department Research University Center for Dental Medicine University of Basel Basel Switzerland; ^4^ Bottmedical AG Technologiepark Basel Basel Switzerland

**Keywords:** antibiotics, antimicrobials, biofilms, cinnamaldehyde, dental aligners, streptococci

## Abstract

**Aims:**

In the context of minor orthodontic intervention using clear aligner technologies, we determined antimicrobial properties of a cellulose‐based material loaded with essential oils such as cinnamaldehyde.

**Methods and Results:**

Isothermal microcalorimetry was used to assess the growth of bacterial biofilms at the interface between the tested material and the solid growth medium. The calorimetric data were analyzed using conventional growth models (Gompertz and Richards), and inhibition at 12 and 24 h was calculated.

**Conclusions:**

The tested material showed antimicrobial properties against *Staphylococcus epidermidis* as well as *Streptococcus mutans* and *Streptococcus mitis* clinical isolates. The inhibition was more pronounced against *S*. *epidermidis*, for which growth rate was reduced by 70% and lag phase was extended by 12 h. For *S*. *mutans* and *S*. *mitis*, the decrease in growth rate was 20% and 10%, and the lag phase increased by 2 and 6 h, respectively.

**Significance and Impact:**

Clear aligners for minor teeth alignment are becoming very popular. As they must be worn for at least 22 h per day for up to 40 weeks, it is important that they remain clean and do not promote caries formation or other oral infections. Therefore, introducing material with antimicrobial properties is expected to maintain oral hygiene during the aligner therapy. Here, we demonstrate the use of cinnamaldehyde for reducing microbial growth and biofilm formation on cellulose‐based dental clear aligners.

## INTRODUCTION

Malocclusion is at least as high in adults as in children and adolescents. In adults, crowding and spacing are among the most common problems (Bilgic et al., 2015; Boyd et al., 2000). Crowding is present in 24% of women and 14% of men. Similarly, spacing is found in 8% of women and 13% of men (Boyd et al., 2000; Buttke & Proffit, 1999). This emphasizes that a rather large fraction of the global population qualifies for minor teeth alignment therapy and the increase in adults seeking orthodontic treatment (Rosvall et al., 2009; Weir, 2017). In addition, there has been a strong demand for those appliances to become both more aesthetic (transparent) and more comfortable (submillimetre‐thin plastic film) than conventional fixed appliances (Weir, 2017). However, for a successful aligner therapy, transparent braces need to be worn as much as possible (i.e. 24 h a day with short removal periods for meals and cleaning) resulting in a significantly increased risk of bacterial growth and subsequent damage to enamel and gingiva. Thus, adding antimicrobial properties to the material used for the braces is considered a promising strategy to limit biofilm formation to further improve the patient's oral hygiene and reduce the risk of caries.

Measuring implant antimicrobial properties is not a simple task. The existing methodology in the International Organization for Standardization (ISO) to determine the antimicrobial activity of plastic surfaces (ISO 22196) is not always applicable because the material should be flat, smooth and available in 50 mm × 50 mm size. This standard also dictates the specific microorganisms to be used for such an evaluation. As a consequence, in many cases, the use of this standard is not possible, especially for materials ‘in development’ or for in situ applications (oral environment for example) where the chosen microorganisms are not relevant. Previous studies have shown that isothermal microcalorimetry is able to accurately determine antimicrobial efficacy of coated implant material providing values close to those obtained from other studies using optical methods where *Staphylococcus epidermidis* biofilms were grown on a transparent plastic polymer (Braissant et al., 2015; Guimond‐Lischer et al., 2016). Calorimetry provides dynamic measurements of metabolic heat, making it possible to gather additional valuable data on the mode of action of the antimicrobial. Indeed, it can help distinguish between bactericidal or bacteriostatic effects (Braissant et al., 2010).

The present study investigates the use of isothermal microcalorimetry to determine the antimicrobial properties of cellulose‐based material loaded with cinnamaldehyde at various concentrations. Cinnamaldehyde is an essential oil that is listed as GRAS (generally recognized as safe) and can be used in food and fragrances (Eilerman et al., 2014). In addition, cinnamaldehyde has antimicrobial and antifungal properties making it interesting for many applications ranging from food processing and packaging to wound dressing (Liu et al., 2017; Manu, 2016; Sanla‐Ead et al., 2012). Considering its properties, it is an appealing compound to make dental appliances antimicrobial and deserves investigation.

## MATERIALS AND METHODS

### Materials

The cellulose‐based material thermofoil (NaturAligner^®^, Bottmedical AG), having a thickness of 550 µm was used. Cinnamaldehyde (trans‐cinnamaldehyde 99%, Sigma‐Aldrich) was used as antimicrobial agent incorporated in the coating. The concentration of cinnamaldehyde was adjusted to the following concentrations per coating surface area: 0.029 ± 0.001 nl·mm^−2^, 0.29 ± 0.01 nl·mm^−2^ and 2.9 ± 0.1 nl·mm^−2^. These tailored aligner material thermofoils have been thermoformed with a pressure‐thermoforming machine (Biostar, Scheu Dental GmbH) at a heating coil temperature of 220°C for a period of 35 s and a pressure of 5 bars over a flat surface based on a 3D model (Formlabs 3 printer with grey resin). Note that cinnamaldehyde has a boiling point of around 251°C and that no loss is expected due to the temperature used. Finally, these thermoformed foils were cut into discs with a diameter of 10 mm to be used in the antimicrobial testing experiments.

### Microorganisms and growth conditions

*S*. *epidermidis* (ATCC 49461) was used as a typical test microorganism (see Pharmacopoeia Europa (Ph. Eur; Schorn, 1993)), and clinically relevant isolates from *Streptococcus mutans* (ATCC 25175) and *Streptococcus mitis* (ATCC 49456) were used as oral microbiome representatives. *S*. *mitis* and *S*. *mutans* are considered as early colonizers of the tooth surface. However, *S*. *mutans* is usually present in lesser amounts in the early plaque biofilm and becomes predominant at later stages (Astasov‐Frauenhoffer & Kulik, 2020; Chenicheri et al., 2017). Still, both are considered relevant as potential early colonizers for aligners used for up to 12 h (i.e. roughly overnight).

Using previously frozen aliquot, precultures of *S*. *epidermidis* were made in Luria Broth (LB) and precultures of streptococci were made in brain–heart infusion (BHI). Bacterial concentrations in the precultures were estimated by plating the different bacteria on their respective medium (i.e. Luria Agar (LA) and agarized BHI (aBHI).

Ten microliters of the preculture were placed on a plastic disk. The inoculated side of the disks was placed facing solid medium (~10 ml of LA or aBHI) previously prepared in 20‐ml microcalorimetric vials. A slight pressure was applied on the backside of the disk until the liquid covered all the surface between the disk and the agar media. The vials were sealed and introduced in a TAM Air (TA instruments) set at a temperature of 37°C at least 2 days before the measurement. Uninoculated samples (LA + uninoculated sterile disk) were also prepared and served as negative controls.

Following the introduction of the samples in the calorimeter and a period of 1 h for thermal equilibration the heat flow was recorded until the signal returned to baseline. Each calorimeter run contained four treated samples and four controls. An additional run was performed to assess the baseline drift of the instrument. This baseline drift was measured using freshly prepared sterile LA medium and was between 0 and 2 µW over 1 week.

### Data analysis and statistical analysis

The heat flow data were extracted as an ASCII file. The heat flow curves were integrated to obtain total heat over time curves. The heat over time data for *S*. *epidermidis* were then fitted using the modified Gompertz model as rearranged by Zwietering et al. (1990; Equation 1) and modified to use the following parameters: *Q*_t_ the heat produced at time *t*, *Q*_max_ the maximum heat, *µ*_max_ the maximum growth rate and *λ*, the duration of the lag phase (Equation 1). Similarly, heat over time data for streptococci showing more asymmetric growth curves were fitted using the modified Richards model as rearranged by Zwietering et al. (1990; Equation 2) and modified to use the same parameters as above. In the case of the modified Richards model, an additional curve shape factor *v* is added (Equation 2).

(1) $$Q_{t}=Q_{\text{max}}\cdot e^{‐e\left[\left(\frac{\mu_{\text{max}}\cdot e}{Q_{\text{max}}}\right)\cdot \left(\lambda ‐t\right)+1\right]},$$

(2) $$Q_{t}=Q_{\text{max}}\cdot {\left\{1+v\cdot e^{\left(1+\nu \right)}\cdot e^{\left[\frac{\mu_{\text{max}}}{Q_{\text{max}}}\cdot \left(1+\nu \right)\cdot \left(1+\frac{1}{\nu }\right)\cdot \left(\lambda ‐t\right)\right]}\right\}}^{\left(\frac{‐1}{\nu }\right)}.$$

In addition, using the average calculated parameters, we estimated the heat after 12 and 24 h and estimated the reduction of heat compared with controls. Twelve and twenty‐four hours were chosen because we assumed that the aligners would be worn for period up to 12 h in a row (roughly overnight). After such period, the aligner is usually cleaned by the user and worn again. We included 24 h as many antimicrobial tests, antibiofilm tests or MIC determinations are performed over 24‐h period, thus allowing potential comparison.

All the calculations and curve fitting were performed using the R language for statistical computing (version 3.5.1 – R Development Core Team, 2018) and the grofit package (Kahm et al., 2010). The parameters were compared using the student test after normality assumptions were verified using the Shapiro–Wilk test. The level of significance for all analyses was *p* < 0.05.

## RESULTS

Tables 1 and 2 show the effect of the antimicrobial added to the cellulose‐based material was visible for all microorganisms, but it was more pronounced for *S*. *epidermidis*. In general, the growth of the microorganisms tested on the treated samples showed a decreased growth rate as well as an increased lag phase duration. The preliminary test performed with *S*. *epidermidis* clearly showed dose‐dependent efficacy. For *S*. *epidermidis* the effect becomes more pronounced with increasing concentration of cinnamaldehyde in the coating. The effect on growth rate, lag phase and heat is statistically significant only at the higher concentrations with a decrease of growth rate from 0.80 to 0.24 h^−1^(−70%), an increase in lag phase duration of 12 h and a decrease in heat produced from 68 to 13 J (−81%; see complete data in Table 1 – representative heat flow curves are shown in Figure S1).

**TABLE 1:** Growth parameters of *Staphylococcus epidermidis* in contact with different cellulose‐based aligner material disks loaded with increasing amounts of cinnamaldehyde and growing on LA medium

Material	Growth rate (*μ*) [h^−1^]	Lag phase (*λ*) [h]	Total heat (*Q*) [J]	*n*
NaturAligner^®^ (controls)	0.80 ± 0.12	13.37 ± 3.49	68.32 ± 26.37	11
NaturAligner^®^ + 29 pl/mm^2^	0.69 ± 0.37	29.42 ± 20.98	37.57 ± 21.22	4
NaturAligner^®^ + 0.29 nl/mm^2^	0.48 ± 0.29	48.81 ± 34.74*	39.44 ± 19.93	4
NaturAligner^®^ + 2.9 nl/mm^2^	0.24 ± 0.32	35.48 ± 14.53	13.38 ± 19.84	4
Blanks (uninoculated agar)	0.00 ± 0.00	0.00 ± NA	0.51 ± 0.15	8

Significant differences (*p* < 0.05) compared with the controls are marked in grey.

Abbreviation: NA, not applicable.

* *p* = 0.06.

**TABLE 2:** Growth parameters of *Streptococcus mitis* and *Streptococcus mutans* in contact with cellulose‐based aligner material with or without cinnamaldehyde and growing on brain–heart infusion medium

Organisms	Material	Growth rate (*μ*) [h^−1^]	Lag phase (*λ*) [h]	Total heat (*Q*) [J]	*n*
*S. mutans*	NaturAligner^®^ (controls)	0.32 ± 0.03	19.34 ± 1.87	34.27 ± 2.08	4
*S. mutans*	NaturAligner^®^ + 2.9 nl/mm^2^	0.26 ± 0.05*	21.99 ± 2.95^†^	31.40 ± 4.82	4
*S. mitis*	NaturAligner^®^ (controls)	0.29 ± 0.01	18.58 ± 2.32	21.47 ± 1.15	4
*S. mitis*	NaturAligner^®^ + 2.9 nl/mm^2^	0.26 ± 0.01	24.69 ± 3.27	24.43 ± 1.10	4
Blanks (sterile)	Uninoculated agar	0.00 ± 0.00	0.00 ± NA	0.51 ± 0.15	8

Significant differences (*p* < 0.05) compared with the controls are marked in grey.

Abbreviation: NA, not applicable.

* *p* = 0.08.

† *p* = 0.11.

The antimicrobial effect was less pronounced against streptococcal species (Table 2 representative heat flow curves are shown in Figure S2). A statistically significant effect was shown only for *S*. *mitis* where a significant reduction of the growth rate coupled with a significant increase in lag phase was observed. Although for *S*. *mitis*, the reduction in growth rate was only from 0.29 to 0.26 h^−1^ (−10%), the increase in lag phase was 6 h, a time that is still of interest when considering the interval between teeth and aligners cleaning. Finally, the total heat released was slightly higher for treated samples of *S*. *mitis*; however, this might be due to higher energy release due to the presence of cinnamaldehyde. Similarly, *S*. *mutans* also showed a reduction of the growth rate and an increase in the duration of the lag phase. The differences were not statistically significant (*µ*_max_: *p* = 0.08, *n* = 8; *λ*: *p* = 0.11, *n* = 8) although the trends are clearly visible.

**FIGURE 1:**
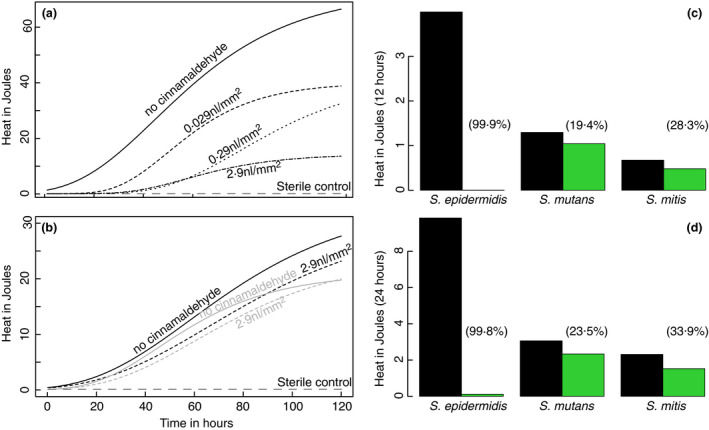
(a) *Staphylococcus epidermidis* growth curves computed from average growth rate (*µ*), lag phase (*λ*) and total heat (*Q*) from Table 1, for uncoated (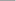) and coated disks with increasing concentration of cinnamaldehyde 0.029 nl mm^−2^ (– – –), 0.29 nl mm^−2^ (‐ ‐ ‐ ‐) and 2.9 nl mm^−2^ (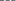). Dark grey long dashes (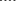) indicate sterile controls. (b) *Streptococcus mutans* (black) *and S*. *mitis* (grey) growth curves computed from average growth rate (*µ*), lag phase (*λ*) and total heat (*Q*) from Table 2, for uncoated (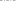 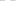) and coated disks with a concentration of cinnamaldehyde 2.9 nl mm^−2^ (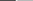). Dark grey long dashes (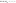) indicate sterile controls. (c, d) show the heat produced after 12 (c) and 24 h (d) for untreated disks (black) and disks with a concentration of cinnamaldehyde 2.9 nl mm^−2^ (green). Numbers in parentheses indicate the percentage of reduction

The average curves (Figure 1) computed with the parameters obtained (Tables 1 and 2) show the lower growth on treated material is visible. Looking at the first 12 and 24 h (possible time intervals between two teeth brushing), the reduction of growth is very high for *S*. *epidermidis* (99.9% after 12 h and 99.8% after 24 h) and still quite important for *S*. *mutans* (19.4% after 12 h and 23.5% after 24 h) and *S*. *mitis* (28.3% after 12 h and 33.9% after 24 h). Although the material does not have the same efficacy against all biofilm formers, it can still delay the growth of biofilm formers and thus decrease the amount of newly formed biofilm between two teeth brushing events.

## DISCUSSION

The use of cinnamaldehyde as an antimicrobial has been widely described, and it is usually recognized as an efficient and safe compound (Doyle & Stephens, 2019; Vasconcelos et al., 2018). Up to now the use of cinnamaldehyde in coatings has been mostly restricted to food packaging (Manu, 2016; Sanla‐Ead et al., 2012), however, application as wound dressing are now being considered (Liu et al., 2017). Its efficacy against oral pathogens such as *S*. *mutans* and *S*. *sobrinus* has been demonstrated previously (Bae et al., 1992; Choi et al., 2016; Didry et al., 1994; He et al., 2019; Ribeiro et al., 2018). Similarly, the antibiofilm properties of this compound have been investigated and demonstrated as well (Albano et al., 2019; He et al., 2019).

To the best of our knowledge, the application of cinnamaldehyde coating on dental aligner material has not been previously described. In this context, our results show that cinnamaldehyde can be used to decrease the rate of growth of microbes on dental aligner as well as delay the biofilm formation with an increase in lag time compatible with the time between two cleanings. Finally, the amount of biofilm formed at the interface between the growth medium and the coating was also less (shown by the lower total heat released). The cinnamaldehyde released from the material was not able to fully inhibit the growth of the pathogen over the rather long (120 h) duration of the measurements. Still, at 12 and 24 h, the reduction in growth was clearly visible in the models as well as raw data (Figure 1; Figures S1 and S2). The effect was clearly marked for *S*. *epidermidis* in comparison with the oral pathogens *S*. *mutans* and *S*. *mitis*. This is not surprising; several studies have shown a high sensitivity of *S*. *epidermidis* to cinnamaldehyde with MIC in the range of 250 to 500 µg·ml^−1^ for both reference strains and clinical isolates (Albano et al., 2019; Firmino et al., 2018). On the contrary, MIC values presented in the literature are scattered with respect to the inhibition of *S*. *mutans*. Indeed, MIC for planktonic *S*. *mutans* range from 100 µg·ml^−1^ to up to 1000 µg·ml^−1^ (Bae et al., 1992; Choi et al., 2016; Didry et al., 1994; He et al., 2019). Similarly, for *S*. *mutans* bactericidal activity of cinnamaldehyde is achieved for concentrations up to 1728 µg·ml^−1^ (i.e. 13 mM) and 2000 µg·ml^−1^ (Choi et al., 2016; Ribeiro et al., 2018).

In addition to its antimicrobial effect, cinnamaldehyde has also been demonstrated to increase the hydrophobicity of some materials, thus potentially decreasing the early adhesion of the biofilm formers and delaying the formation of a mature biofilm (Zodrow et al., 2012). Still, a more complete study involving more strains of streptococci would allow a better estimate of the antimicrobial effect of cinnamaldehyde in cellulose‐based aligner material. This is especially important as there might be greater differences in the susceptibility of the various strains to cinnamaldehyde. Furthermore, the use of a multispecies biofilm model such as the ‘Zurich biofilm model’ would have been of interest in this type of study (Guggenheim et al., 2004; Schmidlin et al., 2013). However, for comparison purposes with other studies and for future development using single strains facilitates comparisons between antimicrobials and products. In a multispecies biofilm, several species with different MIC are found interacting together and the biofilm formation tends to increase their tolerance to antimicrobials making comparison more complex. Still, such approach might be of use at the preclinical stage.

**FIGURE 2:**
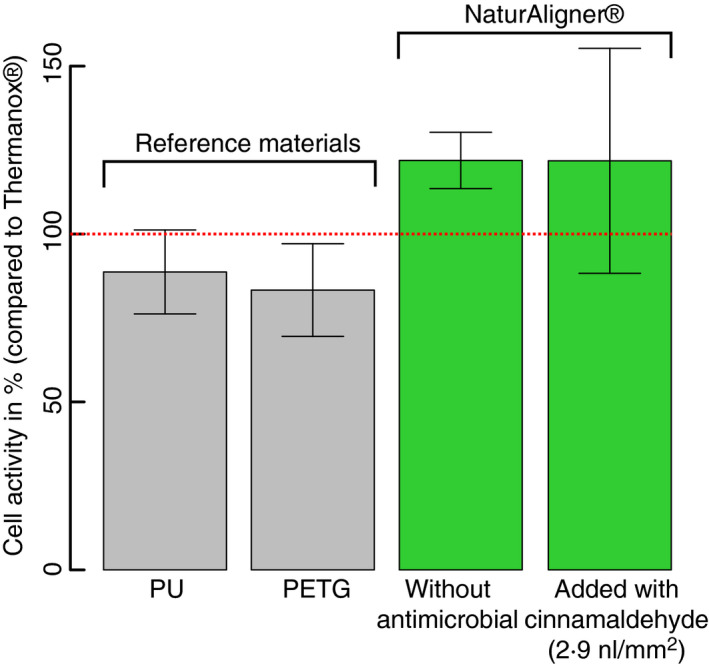
Activity of human primary gingival fibroblast cells after 24 h of growth on reference materials disks (PU and PETG) commonly used in dental appliances and NaturAligner® with and without cinnamaldehyde. All samples are standardized against Thermanox® that is a well‐known biocompatible material

With respect to a material that will remain in the oral cavity for a long period, the toxicity is crucial and must be discussed. Thanks to the previous use for food applications, the toxicological data for oral intake are well known. The oral intake LD_50_ for cinnamaldehyde varies from 2220 mg·kg^−1^ bw in rats to 3400 mg·kg^−1^ bw in guinea pigs. Similarly, the long‐term oral intake ‘*derived no‐ or minimum effect level’* (DN(M)EL), the level of exposure above which a human should not be exposed to a substance is set at 625 µg·kg^−1^ bw·day^−1^ [echa.europa.eu] (https://echa.europa.eu/brief‐profile/‐/briefprofile/100.002.922 – accessed 11/30/2020), about 2 orders of magnitude above the NaturAligner's® agent concentrations of maximum 18 µl per 60 cm^2^ (with a density of 1.05  g·ml^−1^ this is 18.9 µg). This surface (i.e. 60 cm^2^) estimates the upper area of a pair of dental clear aligners. Thus, the cinnamaldehyde concentrations used are unlikely to induce any side effects such as sensitization or allergic reactions. Still, preliminary in vitro results using this specific material (i.e. NaturAligner's^®^) confirmed the absence of negative effects on human primary gingival fibroblast cells (HGF‐1, ATCC American Type Culture Collection) activity compared with other well‐known biocompatible plastics such as Thermanox, polyurethane or polyethylene terephthalate‐glycol (Martina et al., 2019; Figure 2 – complete preliminary testing procedure in Supplementary Material).

An additional advantage of using cinnamaldehyde in dental appliance material is the synergies with many compounds considered in oral care i.g. fluoride and chlorhexidine (Balasubramanian et al., 2020) that are commonly found in toothpaste and mouth washes. Therefore, one can expect that good oral hygiene of a patient and regular use of fluoride toothpaste and mouth rinse is likely to reinforce the antimicrobial effect of the material tested here. In addition, synergistic effects are also observed with conventional antimicrobials such as cefotaxime and ciprofloxacin (Dhara & Tripathi, 2020). This could be useful when considering surgery prior to the use of dental appliances where such antimicrobials could be used (Natarajan et al., 2017). Finally, the good compatibility of cinnamaldehyde with other natural compounds such as carvacrol and eugenol makes it possible to combine such ingredients in the material. This was also visible in our preliminary results combining cinnamaldehyde and limonene (see Figures S3 and S4 for an example). Considering this, cinnamaldehyde appears to be a very good option to render dental aligners antimicrobial. The possibilities of using cinnamaldehyde with other essential oils or other compounds will certainly lead to further development and studies. Those are certainly warranted by the good results obtained in this study.

## CONFLICT OF INTEREST

Tino Töpper and Bekim Osmani are employed by Bottmedical AG.

## Supporting information

Fig S1‐S4
