# Supplementary material for: Cinnamaldehyde as antimicrobial in cellulose‐based dental appliances
Source: J Appl Microbiol. 2021 Sep 22;132(2):1018–24. doi: 10.1111/jam.15283 (PMC9292871; doi:10.1111/jam.15283)
Supplement: Supplementary file 1 — Fig S1‐S4 [file JAM-132-1018-s001.pdf]

SUPPLEMENTARY MATERIAL

Cinnamaldehyde as antimicrobial in cellulose based dental appliances

Sarah Worreth[1,2] , Vivien Bieger[3], Nadja Rohr[3], Monika Astasov-Frauenhoffer[3], Tino Topper[4], Bekim Osmani[4], Olivier Braissant[1,*]

1: Department of Biomedical Engineering, University of Basel, Gewerbestrasse 14, 4123 Allschwil, Switzerland.

2: IUT Nancy-Brabois, Université de Lorraine, Lieu-dit Le Montet, Rue du Doyen Urion, 54600, Villers-lès-Nancy, France.

3: Department Research, University Center for Dental Medicine, University of Basel, Mattenstrasse 40, Basel, Switzerland.

4: Bottmedical AG, Technologiepark Basel, Hochbergerstrasse 60C, 4057 Basel, Switzerland.

*** Corresponding author:** Olivier.braissant@unibas.ch

Preliminary testing of cytotoxicity methodology

Human primary gingival fibroblast cells (HGF-1, ATCC: American Type Culture Collection, Manassas, Virginia, US) have been cultivated in Dulbecco's Modified Eagle Medium (DMEM high glucose, Sigma-Aldrich, Germany) added with 1% L-glutamine (Gibco, Thermo Fisher Scientific), 1% sodium-pyruvate (Gibco, Thermo Fisher Scientific), 1% penicillin-streptomycine (Sigma-Aldrich), 1% amphotericin B solution (Sigma-Aldrich) and 10% fetal calf serum (FCS, bioswisstec, Switzerland). The cultures were incubated for an average of three weeks in the incubator (B5060 EK/CO_2, Heraeus, Hanau, Germany) at 37°C with 5% CO_2, until confluence. During this period the culture medium was changed every second day. Cells were then washed twice with phosphate-buffered saline solution (PBS, Gibco, Thermo Fisher Scientific) without calcium and magnesium and detached using 0.5% trypsin / 0.2% EDTA solution (Sigma-Aldrich). 10^4 cells in a drop of 50µL cell culture medium were seeded per specimen surface in 24-well plates. To prevent cells from growing on well-bottoms, the drops were carefully placed on each specimen. After 1 hour in the incubator, an additional 400µL of cell culture medium was added and cells were cultivated for another 23 hours in the incubator. Polystyrene discs (Thermanox, Faust Laborbedarf, Schaffhausen, Switzerland) were used as control. Similarly, uninoculated triplicate of each samples were also measured served as blanks.

The activity of gingival fibroblasts on each surface was evaluated on 13mm discs using the WST-1 assay (WST-1, Sigma Aldrich) after 24 h. Specimens were rinsed once with PBS and added with 500µL of WST-1 solution mixed with DMEM (1:10) was added to each well and incubated for 2 h. Afterwards, 2x200µL of each supernatant is transferred to another 96-well plate and the optical density (OD) was recorded at 490nm with a micro-plate reader (RT-2100C Microplate Reader, Versamax, Molecular Devices LLC, California, US). Cell viability of 100% is attributed to the cells grown on polystyrene control discs. Relative cell viability is calculated using the following equation:

$$Relative cell viability(\%) = \frac{(OD_{sample} - OD_{blank\,sample})}{(OD_{Thermanox} - OD_{blank\,Thermanox})} * 100$$

Example of raw data and integrated heat over time curve for *S.epidermidis*.

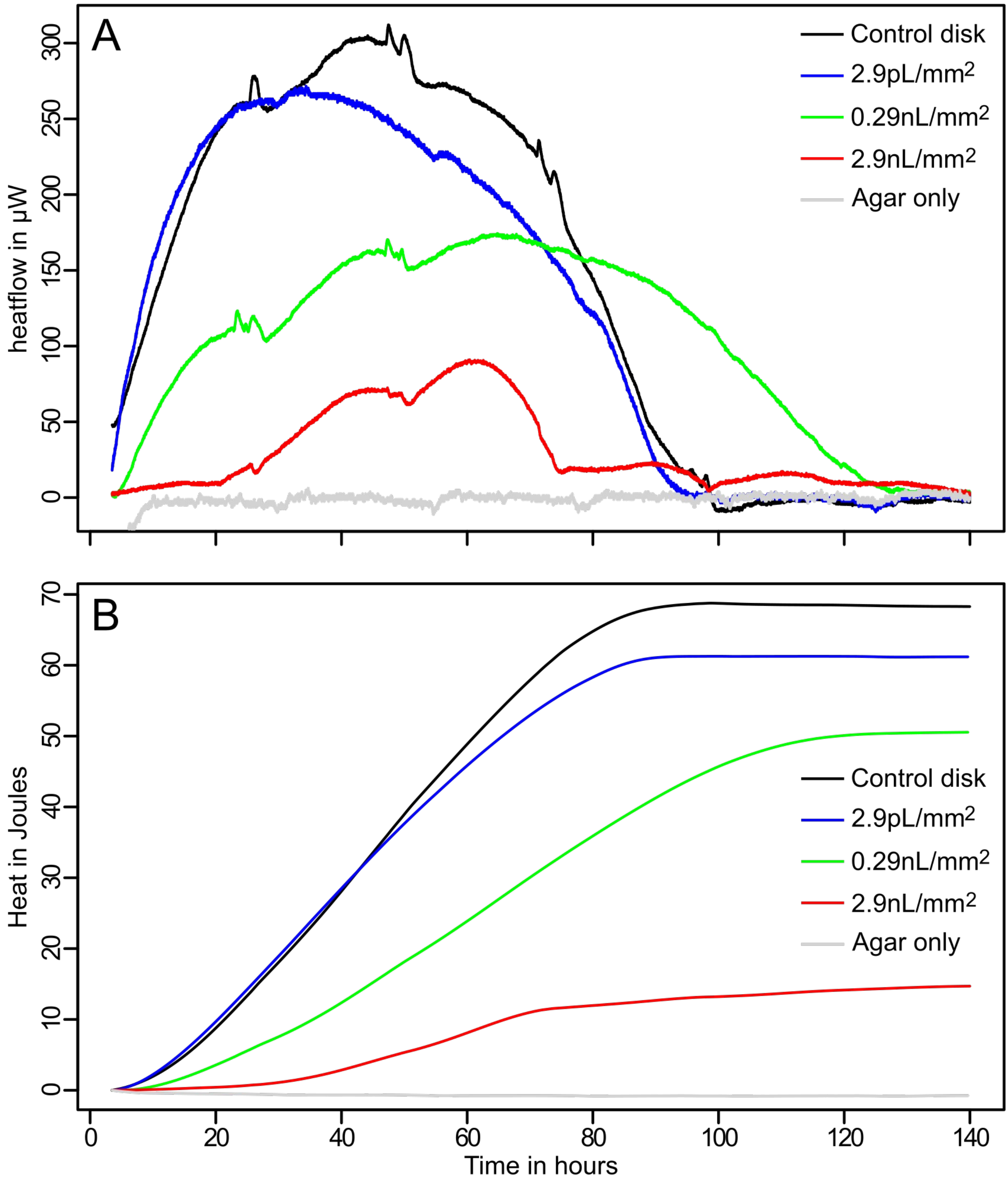

<u>Figure S1:</u> Growth of *Staphylococcus epidermidis* monitored using isothermal microcalorimetry. Raw heatflow data with increasing concentration of cinnamaldehyde (top panel). Note that bumps and spikes in the raw signal are due to strong air conditioning during summer. Integrated heat over time curves derived form the heatflow data. Note that those are only minimally affected by the air-conditioning (bottom panel).

Example of raw data and integrated heat over time curve for streptococci.

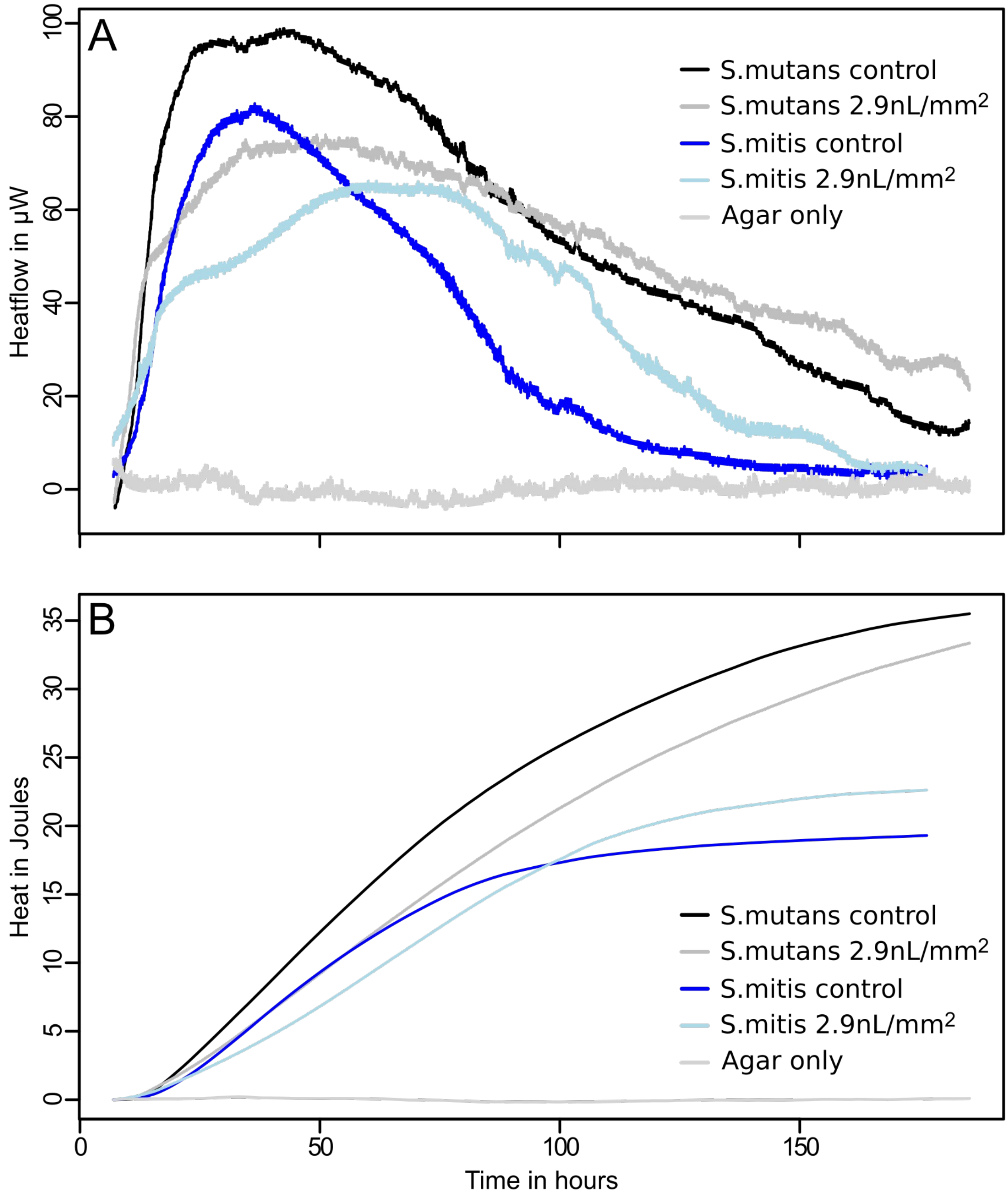

Figure S2: Growth of *Streptococcus mitis* (blue) and *S. mutans* (black) monitored using isothermal microcalorimetry. Raw heatflow data with and without of cinnamaldehyde (top panel). Integrated heat over time curves derived form the heatflow data (bottom panel).

Preliminary results obtained with Limonene

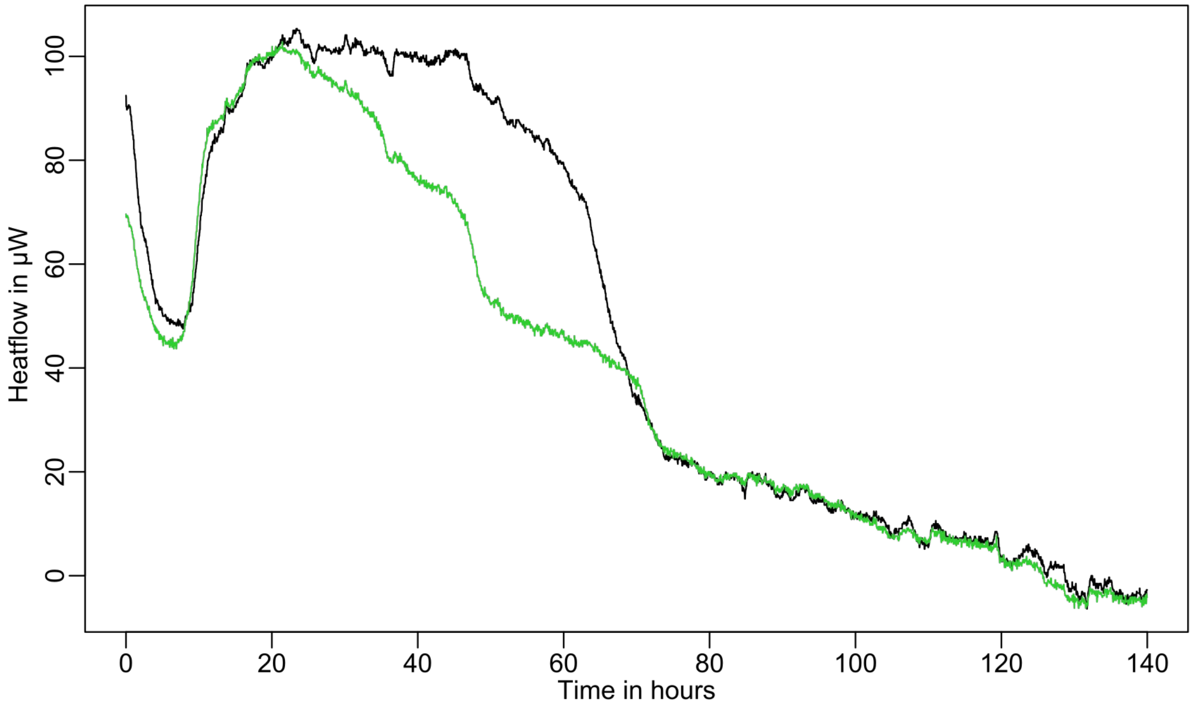

Figure S3: Raw calorimetric data obtained using the methodology described in the main text with discs loaded with cinnamaldehyde and limonene show the effect of these compounds on *Streptococcus mutans*. Black line control disc (no antimicrobial) green line sample disk with cinnamaldehyde and limonene.

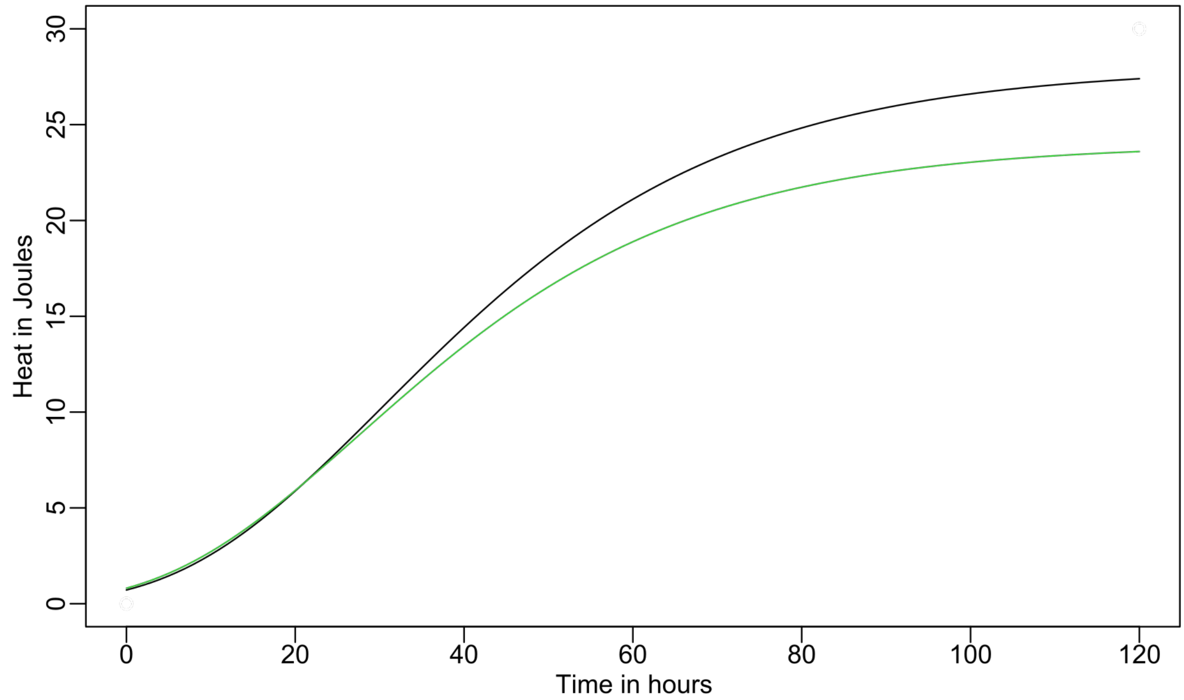

Figure S4: Heat over time curves resulting from the integration of the raw data in figure S1. The heat over time curve is used as a proxy for the growth curve. Black line control disc (no antimicrobial) green line sample disk with cinnamaldehyde and limonene.
